# Intraconal orbital dermoid cyst: a rare location

**DOI:** 10.4322/acr.2021.282

**Published:** 2021-06-25

**Authors:** Swagatika Samal, Mukund Namdev Sable, Sidharth Pradhan, Pradeep Pradhan

**Affiliations:** 1 All India Institute of Medical Sciences, Department of Pathology, Bhubaneswar, Odisha, India; 2 All India Institute of Medical Sciences, Department of ENT and Head Neck Surgery, Bhubaneswar, Odisha, India

**Keywords:** Dermoid cyst, Orbit, Management

## Abstract

Intraconal dermoid cysts are very unusual in routine clinical practice. Clinical symptoms depend upon the site and extension of the lesion. Though rare, proptosis, diplopia, and orbital pain are the presenting symptoms encountered in patients with an intraorbital dermoid cyst. Although radiology can be diagnostic, a complete correlation with the final histopathology is always mandatory for its confirmation. Endoscopic excision of the cyst ensures a complete cure for the disease without any intraoperative/postoperative complications.

## INTRODUCTION

A dermoid cyst is a benign soft tissue lesion that arises from the embryonic epithelium along the embryonic suture lines in the body. It constitutes approximately 2% of all orbital tumors.^1^ It is the commonest intraorbital benign lesion encountered in the pediatric age group, and adults are rarely affected by the disease.^2^ Depending upon the location with respect to the musculofascial cone, it can be extraconal or intraconal, and the occurrence of the cyst in the intraconal space is very rare, accounting for 0.5–0.6% of the total.^3^^,^^4^ Based on the Pub Med database using Intraconal and dermoid cyst as the keywords, only 6 case reports have been described in the literature from the year 1986 to 2020. Three of them involved the intraconal compartment of the orbit,^5^^,^^6^^,^^7^ and in 3 cases it was arising from the lateral rectus muscle.^8^^,^^9^ Herein, we report a large intraconal dermoid cyst presenting with proptosis, diplopia, and orbital pain, compressing the optic nerve in a 30-year-old male who was successfully managed through a transnasal endoscopic approach.

## CASE REPORT

**Figure 1:**
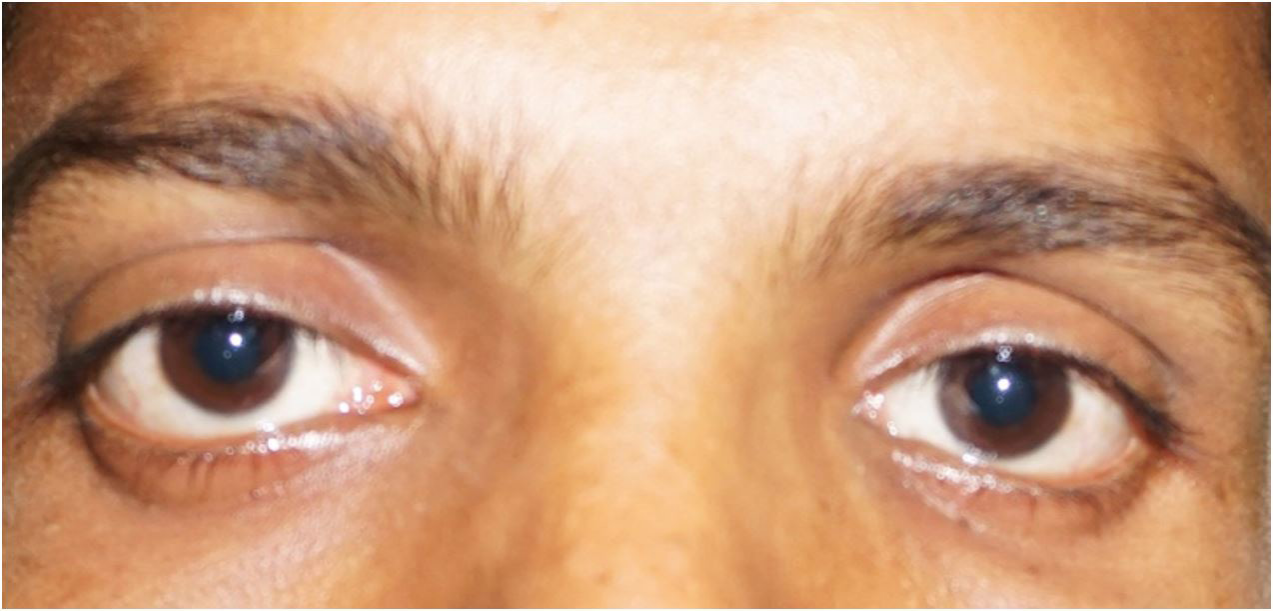
Photograph of the patient showing proptosis of the right eye.

**Figure 2:**
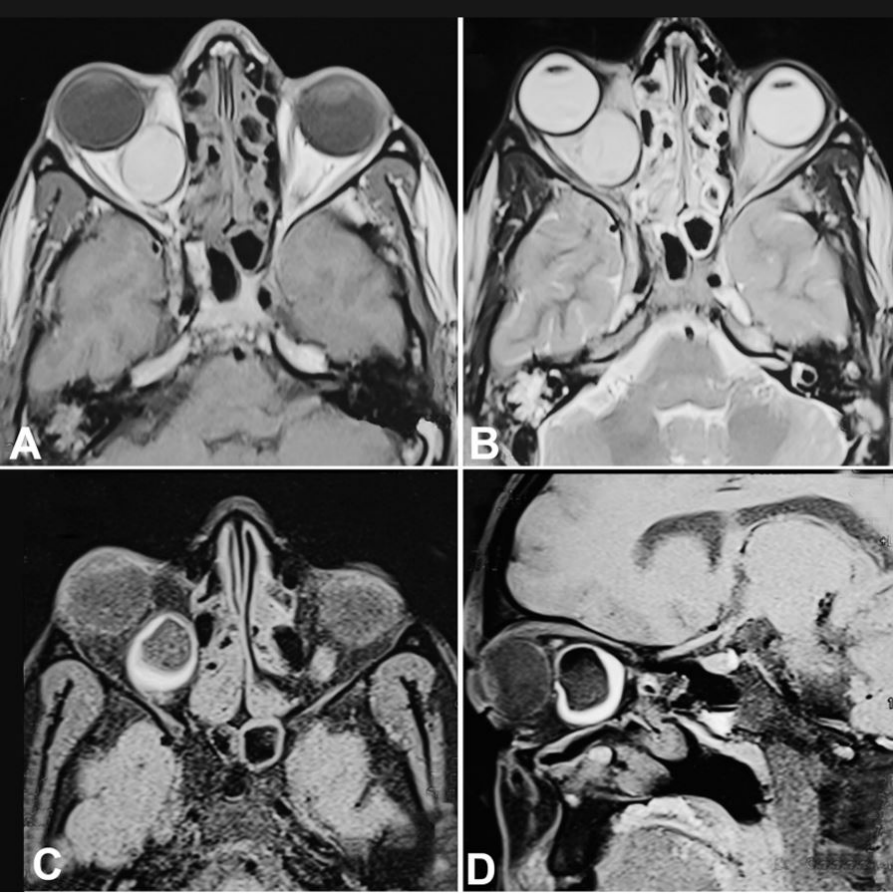
Orbit MRI. The cyst showed a hyperintense signal in both T1 (**A**) and T2-weighted images (**B**); **C** and **D** – T1- weighted image with fat-saturated sequence shows hypointense signals.

A 30-year male presented to the outpatient department with pain and bulging over the right eye for 3 months and diplopia for 2 months (Figure 1). There was no abnormality in the visual acuity. Restriction of eye movement was detected towards the right lateral gaze. The otoscopic examination was found normal. There was no significant neurological deficit. Ophthalmic examination revealed normal visual acuity and field of vision except axial proptosis. The contrast-enhanced MRI scan of the orbit and brain revealed a 3.0 cm well-defined retrobulbar cystic mass occupying the whole intraconal space on the right side, displacing the optic nerve superolateral and the medial rectus in the medial aspect without any intracranial extension. The adjacent bony structures were found intact without any erosion or hyperostosis. On the MRI, the mass was hyperintense in both T1-weighted and T2-weighted images (Figure 2A and 2B). Again, T1- weighted image with a fat-saturated sequence revealed hypointense signals due to the presence of the fat component in the dermoid cyst (Figure 22D).

**Figure 3:**
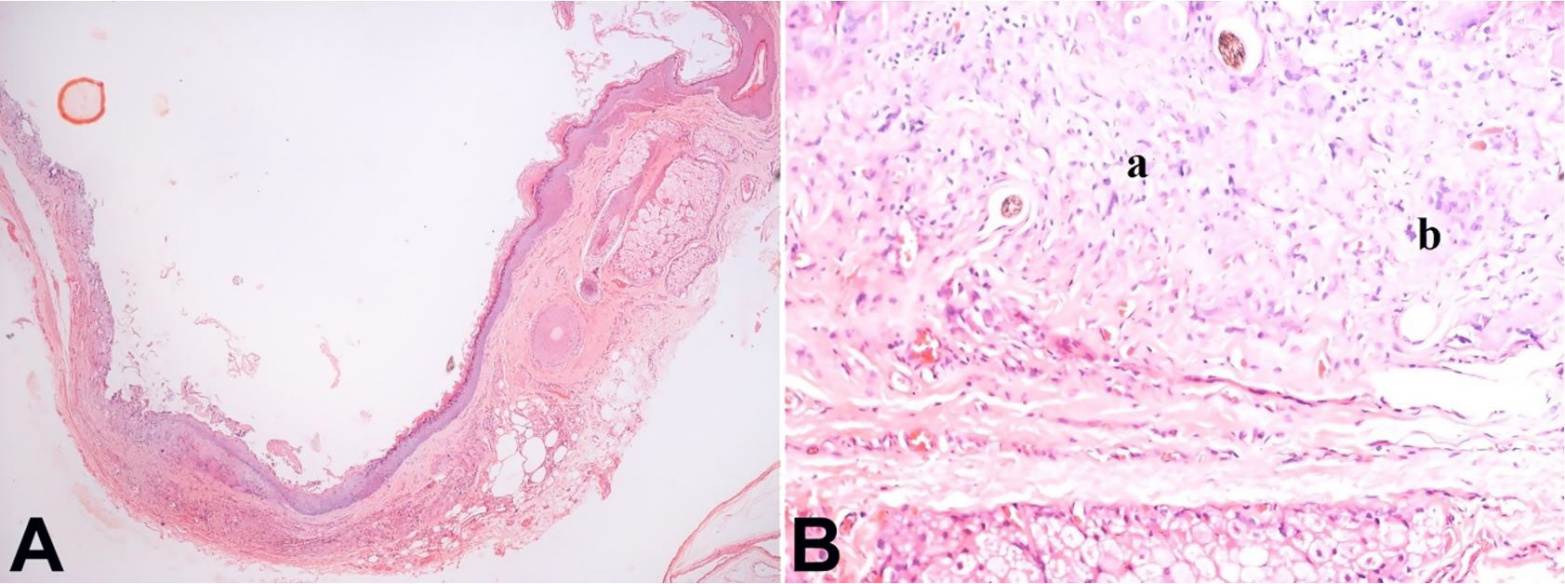
**A** – Photomicrograph of the cystic lesion shows the cyst wall lined by stratified squamous epithelium with keratinization and presence of adnexal structures [H&E 4x]; **B** – Photomicrograph shows part of cyst wall with lymphocytic infiltration (marked a) and focal foreign body giant cell reaction (marked b). [H&E 40x].

**Figure 4:**
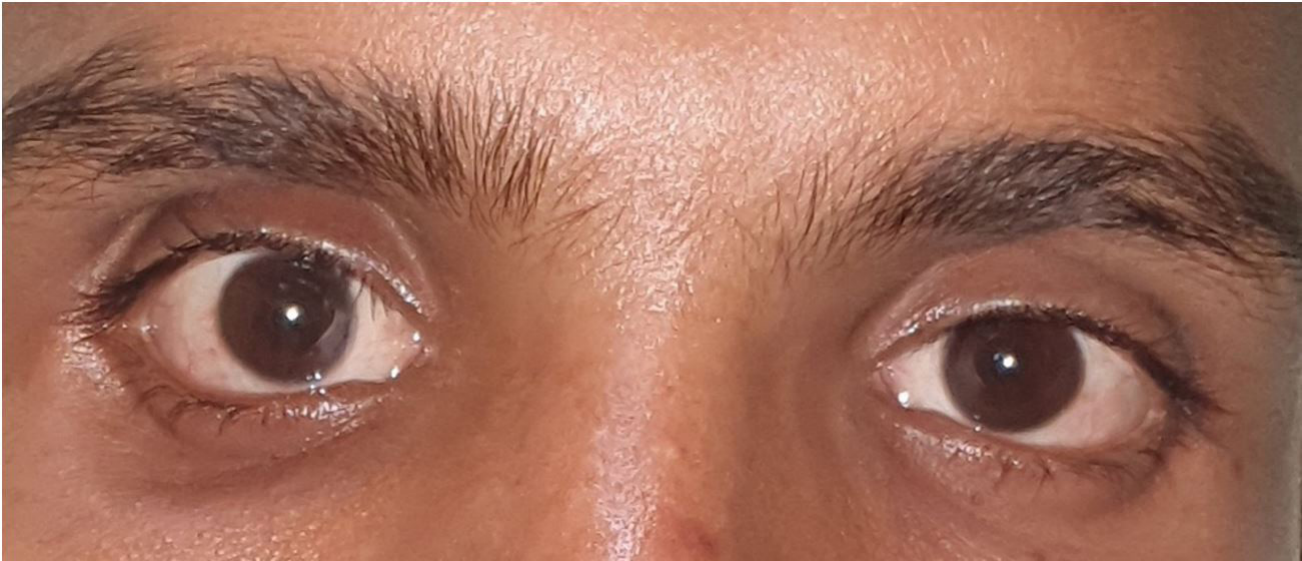
Postoperative photograph of the patient one month after the surgery.

After informed written consent, the patient was planned for excision of the mass through a transnasal endoscopic approach. Endoscopic orbital decompression was performed, the lamina papyracea was removed by a freer’s elevator. The periorbita was completely exposed. A horizontal incision was given over the periosteum, and the medial rectus muscle was incised horizontally and later retracted to expose the whole cyst. The mass was identified and delineated all around from the extraocular muscles and the optic nerve and later removed completely by gentle blunt dissection and sent for histopathological examination for confirmation. On histopathology, a cyst wall was identified and was lined by keratinized stratified squamous epithelium with the presence of adnexal structures (Figure 3A). There was lymphocytic infiltration with a focal giant cell reaction without any evidence of granuloma or malignancy (Figure 3B). The intraoperative and postoperative events were found favorable. The patient is on regular follow-up for the past six months with normal ocular motility without radiological/clinical recurrence of the disease (Figure 4).

## DISCUSSION

The occurrence of a complete intraorbital and intraconal dermoid cyst is extremely unusual in clinical practice. Based on the Pub Med database using intraconal and dermoid cyst as the keywords, only 6 case reports have been described in the literature. Three of them involved the intraconal compartment of the orbit,^5^^,^^6^^,^^7^ and 3 cases arose from the lateral rectus muscle.^8^^,^^9^ At present, we have reported a large intraorbital dermoid, which was completely occupying the intraconal compartment in the right eye. In the majority of the cases, the orbital dermoids are located in the superficial compartment of the eye, usually detected in the childhood period with a painless periorbital swelling as the predominant complaint by most of the patients. In contrast, in the present case, the dermoid cyst was found in an unusual location, i.e., the deep cone of the orbit in an adult patient, which was again very unusual. Clinical symptoms of the orbital dermoid depend upon the site and size of the lesion with respect to the involvement of the orbital compartment. Majority of the cases are asymptomatic except for a painless periorbital swelling because of their superficial location. Dermoid cyst with intraconal location can have variable presentation depending upon the amount of lateral pressure exerted by the mass to the vital neuromuscular structures.^2^^,^^3^ As it was evident in the present case, the patient presented with proptosis and diplopia with restriction of the right lateral movement of the eye, which could be due to lateral pressure over the extraocular muscles.

The intraocular pain could be due to the inflammatory reaction in the dermoid cyst, which could be due to the presence of chronic inflammation, as demonstrated by Abou-Rayyah et al.^10^ confirmed after the histopathological and radiological examination. Again, the local inflammation could be due to the tissue reaction induced by the leakage of the keratinous material or the lipids from the cyst^11^. Radiology is considered as the mainstay of diagnosis for the orbital dermoid, as fine needle aspiration is not always possible, especially in the deep-seated mass where the chance of neurovascular damage is always a risk. In contrast-enhanced CT scan, it appears as a well-defined cystic lesion with hyperostosis and peripheral rim calcification.^3^^,^^4^^,^^12^^,^^13^ Contrast-enhanced MRI reveals the presence of the fat component and better resolution of the soft tissue. MRI is superior to the CT scan in finding the detailed anatomy of the lesion, including the extension and spatial orientation of the surrounding neuromuscular structures and the intercanal connection to the orbital rim. As evident in the present case, the mass was causing a lateral pressure over the right lateral rectus muscle, and the optic nerve, without causing any damage to the nerve in the preoperative period. Along with the primary diagnosis, MRI is quite helpful in the detection of the complications, especially the hemorrhage and rupture in the cyst. Although any orbital lesion can be a differential diagnosis of a dermoid cyst, a dependable diagnosis can be obtained by correlating the radiological and pathological findings. Surgical excision is the definitive treatment of the intraconal dermoid where lesions are removed by orbital incision.^14^^,^^15^ With the advancement of endoscopic sinus surgery, this can be successfully excised by a transnasal endoscopic approach without causing significant intraoperative/postoperative morbidities.

## CONCLUSION

Intraconal dermoid is very unusual in the routine practice. Clinical symptoms depend upon the site and extension of the lesion. Though rare, proptosis, diplopia, and orbital pain are the presenting symptoms encountered in patients with intraorbital dermoid. Although radiology can be diagnostic, a complete correlation with the final histopathology is always mandatory. Endoscopic excision of the cyst ensures a complete cure for the disease without any intraoperative/postoperative complications.
